# Correction: Specific Reactions of Different Striatal Neuron Types in Morphology Induced by Quinolinic Acid in Rats

**DOI:** 10.1371/journal.pone.0096966

**Published:** 2014-04-29

**Authors:** The PLOS ONE Staff

## Notice of Republication

This article was republished on April 14th, 2014, due to the figures being ordered incorrectly in the previously published article. Please download the PDF again to view the corrected article. The originally published, uncorrected article and the republished, corrected article are provided here for reference.

## Supporting Information

File S1 Originally published, uncorrected article.
(PDF)

File S2 Republished corrected article.
(PDF)
