# Supplementary material for: Correction: Specific Reactions of Different Striatal Neuron Types in Morphology Induced by Quinolinic Acid in Rats
Source: PLoS One. 2014 Apr 29;9(4):e96966. doi: 10.1371/journal.pone.0096966 (PMC4004569; doi:10.1371/journal.pone.0096966)
Supplement: File S2 — Republished corrected article. (PDF) [file pone.0096966.s002.pdf]

OPEN ACCESS Freely available online PLOS ONE

Specific Reactions of Different Striatal Neuron Types in Morphology Induced by Quinolinic Acid in Rats

Qiqi Feng[1,2], Yuxin Ma[1,3], Shuhua Mu[1], Jiajia Wu[1], Si Chen[1], Lisi OuYang[1], Wanlong Lei[1]*

1 Department of Anatomy, Zhongshan School of Medicine, Sun Yat-sen University, Guangzhou, China, 2 Department of Nephrology, The Third Affiliated Hospital of Sun Yat-sen University, Guangzhou, China, 3 Department of Anatomy, School of Basic Medicine, Guangdong Pharmaceutical University, Guangzhou, China

Abstract

Huntington's disease (HD) is a neurological degenerative disease and quinolinic acid (QA) has been used to establish HD model in animals through the mechanism of excitotoxicity. Yet the specific pathological changes and the underlying mechanisms are not fully elucidated. We aimed to reveal the specific morphological changes of different striatal neurons in the HD model. Sprague-Dawley (SD) rats were subjected to unilaterally intrastriatal injections of QA to mimic the HD model. Behavioral tests, histochemical and immunhistochemical stainings as well as Western blots were applied in the present study. The results showed that QA-treated rats had obvious motor and cognitive impairments when compared with the control group. Immunohistochemical detection showed a great loss of NeuN+ neurons and Darpp32+ projection neurons in the transition zone in the QA group when compared with the control group. The numbers of parvalbumin (Parv)+ and neuropeptide Y (NPY)+ interneurons were both significantly reduced while those of calretinin (Cr)+ and choline acetyltransferase (ChAT)+ were not changed notably in the transition zone in the QA group when compared to the controls. Parv+, NPY+ and ChAT+ interneurons were not significantly increased in fiber density while Cr+ neurons displayed an obvious increase in fiber density in the transition zone in QA-treated rats. The varicosity densities of Parv+, Cr+ and NPY+ interneurons were all raised in the transition zone after QA treatment. In conclusion, the present study revealed that QA induced obvious behavioral changes as well as a general loss of striatal projection neurons and specific morphological changes in different striatal interneurons, which may help further explain the underlying mechanisms and the specific functions of various striatal neurons in the pathological process of HD.

Citation: Feng Q, Ma Y, Mu S, Wu J, Chen S, et al. (2014) Specific Reactions of Different Striatal Neuron Types in Morphology Induced by Quinolinic Acid in Rats. PLoS ONE 9(3): e91512. doi:10.1371/journal.pone.0091512

Editor: Gilles J. Guillemin, Macquarie University, Australia

Received December 2, 2013; **Accepted** February 10, 2014; **Published** March 14, 2014

Copyright: © 2014 Feng et al. This is an open-access article distributed under the terms of the Creative Commons Attribution License, which permits unrestricted use, distribution, and reproduction in any medium, provided the original author and source are credited.

Funding: This research was supported by the National Science Foundations of China (No. 31070941, No. 30770679) and the Major State Basic Research Development Program of China (973 Program, No. 2010CB530004). The funders had no role in study design, data collection and analysis, decision to publish, or preparation of the manuscript.

Competing Interests: The authors have declared that no competing interests exist.

* E-mail: leiwl@mail.sysu.edu.cn

These authors contributed equally to this work.

Introduction

Huntington's disease (HD) is an inherited neurodegenerative disorder characterized by abnormal involuntary movements and cognitive impairment [1]. The pathological hallmark of HD is the selective neuron death in the striatum – loss of spiny projection neurons and relative sparing of aspiny interneurons [2]. The pathogenesis of HD critically involves the mutant gene huntingtin (htt) which encodes a large protein (350 kDa) with a polyglutamine stretch [3,4]. The extent of the polyglutamine expansion is correlated with the severity of symptoms, such as age of onset [5]. Despite the discovery of htt, the pathophysiology of HD and the mechanisms accounting for the selective neuron death still remain unclear. It has been suggested that excitotoxicity, mitochondrial abnormalities and transcriptional dysregulation are some important mechanisms in the progress of HD [6,7,8]. Therefore, the excitotoxin quinolinic acid (QA), mitochondrial toxin 3-nitropropionic acid (3NP) and transgenic models are used to study the pathophysiology of HD [9,10,11].

The "excitotoxicity hypothesis" of neurodegeneration has been proposed for several decades and it persists as a likely pathophysiological mechanism in HD [6,12,13]. The excitotoxin QA has been used extensively to establish HD models in animals since it generally spares aspiny interneurons, relative to spiny projection neurons, which is more analogous to the neuropathology of HD than other excitotoxins such as kainic acid and ibotenic acid [9,14]. Furthermore, it has been demonstrated that QA is a brain endogenous excitotoxin produced and released by infiltrating macrophages and activated microglia, and acts as a neurotoxin, gliotoxin, proinflammatory mediator, pro-oxidant molecule and can alter the integrity and cohesion of the blood-brain barrier [15]. Nevertheless, the issue about the resistance of different striatal neuron types in HD is still controversial. Histologically, the striatum consists of projection neurons (90%–95% in rodents and about 80% in primates) and interneurons (5%–10% in rodents and possibly up to 20% in primates) which are subdivided into four types: parvalbumin (Parv)+, calretinin (Cr)+, neuropeptide Y/somatostatin/neuronal nitric oxide synthase (NPY/SS/nNOS)+ and choline acetyltransferase (ChAT)+ interneurons [16]. A great amount of research applying different HD models concordantly revealed that the striatal projection neurons were vulnerable [11,17,18,19]. However, different studies provided different observations about the preservation of different striatal interneuron types in HD. For instance, plenty of evidence indicated that

the striatal NPY+ interneurons were relatively spared in HD models [17,20,21,22] while several previous studies reported no such sparing [23,24]. In addition, most studies on striatal neurons in HD models mainly focused on the neuron abundance rather than the morphology. In fact, Mu *et al* revealed that the NPY+ and Cr+ interneurons were presented with an increase of fibers and varicosities in the 3NP-induced HD model [25]. It is reasonable to presume that clarifying the specific reactions of different striatal neurons in HD may help further understand the pathophysiology of HD.

The present study aimed to provide more comprehensive evidence for the QA-induced HD model in respect of behavior and histology, so as to help further comprehend the underlying pathophysiological mechanisms of HD. A series of behavioral tests, histological techniques and Western blots were applied to assess the motor and cognitive impairments, the striatal histopathological changes as well as expression levels of different marker proteins for different striatal neuron types.

Materials and Methods

Animals and experimental design

Thirty adult male Sprague-Dawley (SD) rats weighing 250–300 g (obtained from the Center for Experimental Animals of Sun Yat-sen University) were used for this study. All the animal experiments strictly adhered to the Regulations for the Administration of Affairs Concerning Experimental Animals, the Chinese national guideline for animal experiment, issued in 1988. All procedures involving animals and their care in this study were approved by the Animal Care and Use Committee of Sun Yat-sen University (Permit Number: SCXK GUANGDONG 2011-0029). All efforts were made to reduce the number of animals used. The animals were housed in an air-conditioned room under an even light-dark cycle, with food and water *ad libitum.*

The rats were randomly assigned to the QA group ($n = 10$), the control group ($n = 10$) and the normal group ($n = 10$). All rats in the QA group were injected unilaterally into the right striatum with 1 µl 100 mM QA (Sigma) [20] while animals in the control group received 1 µl vehicle, and rats in the normal group did not receive any surgery or treatment. Five days after surgery, all rats were given behavioral tests for five consecutive days. Following a ten-day survival period, fifteen animals (five from each group) were subjected to histological assessments and the rest of them were subjected to Western blots as described below.

Animal surgery

Rats in the QA group were anesthetized with ketamine (150 mg/kg). They were then positioned in a small animal stereotaxic frame (Kopf) and given unilateral injections of 1 µl QA which was dissolved in 10 mM phosphate-buffered saline (PBS) at pH 7.4 into the right side of the striatum at the following coordinate: AP = +1.0 mm; ML = 2.5 mm from bregma; DV = −5.5 mm from dura according to the atlas of Paxinos and Watson [26]. Injections were manually delivered by a Hamilton microsyringe over a period of 10 min, followed by a 5-min delay before the needle was withdrawn. The needle was withdrawn slowly over a period of additional 2 min to prevent diffusion along the needle track. Rats in the control group were treated with the same procedure but received only vehicle. Postoperatively, animals were individually placed in separate cages with free access to food and drink until they recovered from anesthesia. Five days after the surgery, animals were tested by a series of behavioral tasks.

Behavioral tests

Balance beam test. The balance beam test was carried out for rats in all groups three times a day for five consecutive days according to Shear's methods [17]. The rats were trained to travel across a suspended narrow beam (100 cm in length, 7 cm in width, 100 cm elevated above the horizontal surface of the ground) into a dark box (24.5×20×18 cm) at the other end. The completion time (the interval between the moment the rat was released to the moment it entered the dark box) and the number of paw slipping (the forelimb descending more than 1.5 cm below the surface of the beam) were recorded by two observers that were blinded to animal conditions. Animals were given 3 min to complete a trial. If the rat fell down or took more than 3 min, this trial was recorded as incomplete.

Grip strength test. Grip strength test [17] was measured three times a day for five consecutive days. Examiners were blinded to animal conditions. Grip strength was measured by recording the length of time the rat was able to hold on a steel wire (2 mm in diameter, 35 mm in length) suspended 50 cm above the horizontal surface of the ground.

Water maze task. All rats were trained with the learning trial four times a day for five consecutive days, followed by the probe trial on the last day in the water maze task [27,28]. In the learning trials, the target platform was located in different spatial locations across trials, but the visual pattern of the ball which served as a cue was consistent. During each trial, rats were released from four assigned starting points (N, S, E, W) and allowed to swim until they reached the platform within 2 min. The rats were allowed to stay on the platform for 30 sec once they reached the platform or if they failed within 2 min. The latency of reaching platform was recorded. The probe trial was administered immediately following the last learning trial. In the probe trial, the platform was removed from the tank. The animals were released into the tank and allowed to swim for 2 min, and the number of target site crossovers was recorded. The tracks were recorded by a camera and Ethovision software (Noldus, Holland) which recorded the latency of reaching platform and calculated the number of target site crossovers. This task could be acquired by learning an approach response to the visual cue, which was believed to be associated with the mnemonic functions of the striatum.

Histochemical and immunohistochemical methods

Nissl staining. After the five-day behavioral tests, five rats from each group were sacrificed for histochemical and immunohistochemical examinations. Before the following procedures, animals were deeply anesthetized with 10% chloral hydrate (350 mg/kg), then perfused with 300 ml 0.9% saline followed by 400 ml 4% paraformaldehyde in 0.1 M phosphate buffer (PB, pH 7.4, 4°C). Brains were quickly removed and post-fixed overnight at 4°C, and were sliced into coronal sections (30 µm) on a vibratome (VIBRATOME, #053746). Sections were stained with Nissl staining according to previous classic methods [29].

Immunohistochemistry procedures. Brain sections were pre-treated with 0.3% H_2O_2 in 10 mM PBS (pH 7.4, 4°C) for 30 min. Separate series of sections were respectively incubated at 4°C for 48 h with one of the following primary antibodies: mouse anti-NeuN (1:500, Millipore), rabbit anti-Darpp32 (1:200, Cell Signaling, Danvers, MA), mouse anti-Parv (1:1,000, Sigma), rabbit anti-Cr (1:2,000, Millipore), rabbit anti-NPY (1:5,000, ABCAM) and rabbit anti-ChAT (1:1,000, Millipore). After rinsed in 10 mM PBS for three times (5 min/time), the sections were applied with secondary antibodies anti-mouse IgG or anti-rabbit IgG (both 1:200, Sigma) at room temperature for 4 h, followed by three rinses (5 min/time) in 10 mM PBS and incubation with homologous

Table 1. Measures of behavioral and histological tests in the normal group and the control group.

Test	Parameter	Norm	Cont	*p* #
Balance beam test	Number of paw slipping	0.15±0.10	0.17±0.16	0.933
	Completion time (sec)	18.67±4.67	19.45±7.21	0.869
Grip strength test	Hang time (sec)	11.66±3.51	11.47±4.43	0.904
Water maze test	Latency (sec)	37.09±6.30	41.42±10.77	0.558
	Number of target site crossovers	10.80±2.78	11.20±1.93	0.723
Nissl staining	Cell count (/mm^2)	3224.00±365.04	3175.00±207.76	0.772
NeuN labeling	Cell count (/mm^2)	2458.60±96.80	2471.20±119.13	0.917
Darpp32 labeling	Cell count (/mm^2)	2007.20±56.85	1941.80±102.22	0.188
Interneuron number	Parv+ (/mm^2)	79.48±1.79	78.86±2.72	0.783
	Cr+ (/mm^2)	65.76±2.35	65.84±1.92	0.959
	NPY+ (/mm^2)	49.20±1.48	48.90±1.95	0.828
	ChAT+ (/mm^2)	69.34±2.59	69.60±1.55	0.899
Fiber density	Parv+ (/100 μm)	13.04±0.97	13.02±0.96	0.978
	Cr+ (/100 μm)	6.97±0.34	7.04±0.56	0.943
	NPY+ (/100 μm)	5.98±0.40	6.03±0.75	0.926
	ChAT+ (/100 μm)	3.06±0.24	3.16±0.40	0.777
Varicosity density	Parv+ (/100 μm)	4.36±0.71	4.04±0.79	0.599
	Cr+ (/100 μm)	6.98±1.05	7.18±1.63	0.875
	NPY+ (/100 μm)	6.22±0.79	5.80±1.10	0.540
Western blots	NeuN (OD)	1.29±0.08	1.31±0.04	0.493
	Darpp32 (OD)	1.28±0.07	1.28±0.05	0.822
	Parv+ (OD)	1.19±0.10	1.21±0.06	0.634
	Cr+ (OD)	0.67±0.08	0.68±0.05	0.731
	NPY+ (OD)	1.13±0.10	1.13±0.11	0.971
	ChAT+ (OD)	0.48±0.05	0.49±0.02	0.733

Note: values expressed as group means±SD; # normal (Norm) v.s. control (Cont), one-way ANOVA and Fisher's *post hoc* PLSD test.
doi:10.1371/journal.pone.0091512.t001

peroxidase-antiperoxidase (PAP) complex (1:200, Sigma) at room temperature for 2 h. The peroxidase reaction was performed using 3, 3′-diaminobenzidine (DAB, 0.05% in 10 mM PBS, pH 7.4, Sigma) for 2–8 min, and then the sections were mounted onto gelatin-coated slides, routinely dehydrated, cleared and covered with neutral balsam for microscopic detection.

TUNEL assay. To assess apoptotic level of striatal neurons after experimental treatments, immunofluorescent detection of neurons combined with the terminal deoxynucleotidyl transferase dUTP nick labeling (TUNEL) was performed. For the present study, sections were incubated with the primary antibody mouse anti-NeuN (1:500, Millipore) at 4°C for 48 h, and subsequently with rhodamine-conjugated goat anti-mouse IgG (1:200, Jackson ImmunoResearch) at room temperature for 2 h. All sections were thereafter rinsed three times (5 min/time) in 10 mM PBS and performed with TUNEL assay (In Situ Cell Death Detection Kit, POD, Roche) according to the manufacturer's instructions. Sections were mounted on gelatin-coated slides, routinely dehydrated, cleared and covered with glycerol. Section detection and image capture were conducted on a fluorescence microscope.

Western blots

After the five-day behavioral tests, the other five rats from each group were sacrificed for Western blots. Animals were deeply anesthetized with 10% chloral hydrate (350 mg/kg), perfused with 0.9% saline, and then got decapitated. The right striatum was extracted from the brain, and then stored at −80°C before use. Western blots were carried out as previously described [25]. In brief, the tissue was homogenized in a freshly prepared lysis buffer with protease inhibitors, and then centrifuged at 12000 r/min for 30 min. The protein concentration of the homogenate was determined using the BioRad DC protein assay (BioRad Laboratories). 40 μg of total protein from each sample were subjected to an SDS–PAGE gel (10%) and transferred to a PVDF membrane (Millipore). The membrane was blocked with 5% skim milk at room temperature for 2–4 h and incubated at 4°C overnight with one of the following primary antibodies: mouse anti-NeuN (1:1000, Millipore), rabbit anti-Darpp32 (1:250, Cell Signaling), mouse anti-Parv (1:1000, Sigma), rabbit anti-Cr (1:5000, Millipore), rabbit anti-NPY (1:6000, Abcam), rabbit anti-ChAT (1:2000, Millipore) and mouse anti-β-actin (1:2000, Millipore). After washed with TBST for four times (5 min/time), the membrane was incubated with homologous HRP-conjugated secondary antibodies (1:3000, Amersham Biosciences, GE Healthcare) at room temperature for 2 h, and washed again in TBST for four times (5 min/time). Blots were visualized in enhanced chemiluminescence (ECL) solution (Pierce) for 5 min and exposed to hyperfilms (Kodak) for 1–15 min.

Data collection and statistical analysis

Quantification of neuron number, fiber density and varicosity density. The investigators were blinded to which

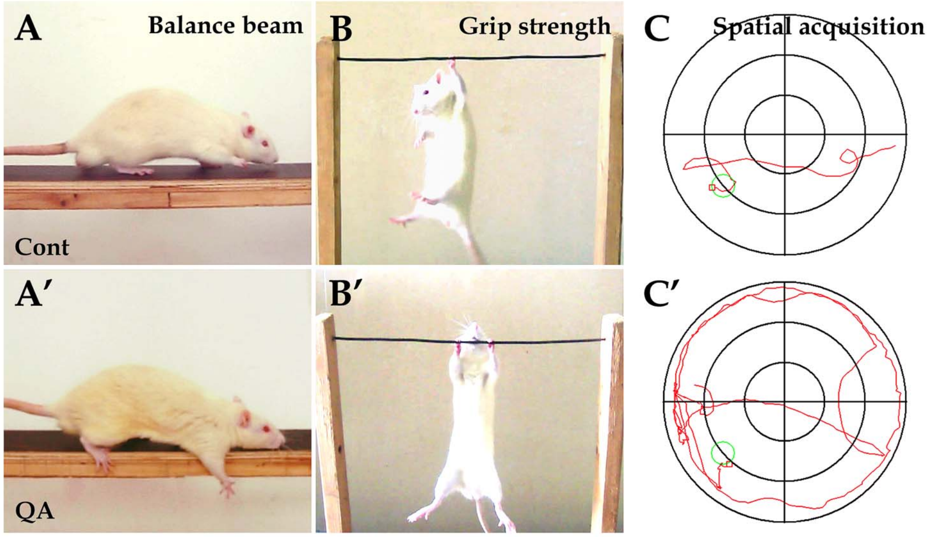

Figure 1. Experimental tests for behavioral deficits induced by QA. In the balance beam test, the control rats were able to travel across the beam easily (A) while rats in the QA group had difficulty in passing across the beam with paw slipping (A′). In the grip strength test, the control rats held the wire firmly (B) while the QA-treated rats manifested hypertonia (B′). In the water maze task, the swim track of rats in the QA group was bending and often along the maze wall (C′) in comparison of the control rats (C). Cont is short for control.
doi:10.1371/journal.pone.0091512.g001

group the sections belonged to when conducting the measurement. The quantification was carried out on every eighth section of the striatum (2.0 mm anterior and 0.7 mm posterior to bregma, ten to eleven sections per animal for each staining method) and the numbers of neurons, fibers and varicosities were counted throughout the depth of the section. In the Nissl-stained sections, the approximate annular zone with 50%–90% neuronal survival around the lesion core was considered the transition zone in the present study. The section was first examined under the light microscope (Olympus BHS) equipped with a camera lucida at 100× magnification, and the center of the lesion core was located which was apparent due to its severe neuron loss. Then the lesioned striatum was divided into 0.1 mm×0.1 mm zones respectively parallel and perpendicular to the ventricular edge of the ipsilateral striatum through the center of the lesion core that was just located. For convenience, an acetate overlay with the zones drawn on it was created for each case to delineate the striatal regions to be counted for each zone. The number of Nissl-stained neurons in each zone was counted at 400× magnification and neuronal survival was expressed as the percentage of neuronal abundance found in the matching zone in control animals. Zones showing 50%–90% survival of Nissl-stained neurons (i.e., zones superior, inferior, medial and lateral to the lesion core with 50%–90% neuronal survival) were considered to be within the transition zone. For each drawn lesioned striatum with Nissl staining, two to three zones in each direction (i.e., superior, inferior, medial and lateral to the lesion core) fell within the transition zone. All the quantification of neuron number, fiber density and varicosity density was performed in these zones. The NeuN-, Darpp32-, TUNEL/NeuN-, Parv-, Cr-, NPY- and ChAT-stained sections adjacent to the corresponding Nissl-stained section were also analyzed. The accurate number of neurons was counted in the NeuN-, Darpp32-, TUNEL/NeuN-, Parv-, Cr-, NPY- and ChAT-stained sections. The quantification of fiber density was performed in the Parv-, Cr-, NPY- and ChAT-stained sections, and the numbers of intersecting processes along a 100-μm length were counted and averaged as the fiber density. The varicosity density was measured in the Parv-, Cr- and NPY-stained sections, and the numbers of varicosities along a 100-μm length were counted and averaged as the varicosity density. The final neuron number, fiber density and varicosity density of each staining method for every rat were the average of the numbers acquired from the ten to eleven equidistantly sampled sections, respectively.

Table 2. Measures of behavioral tests.

Test		Cont	QA
Balance beam test	Number of paw slipping	0.17±0.16	0.89±0.92*
	Completion time (sec)	19.45±7.21	40.36±16.48*
Grip strength test	Hang time (sec)	11.47±4.43	16.45±2.12*
Water maze test	Latency (sec)	41.42±10.77	71.53±25.34*
	Number of target site crossovers	11.20±1.93	2.10±2.69*

Note: values expressed as group means±SD; * $p<0.01$ v.s. control (Cont), one-way ANOVA and Fisher's *post hoc* PLSD test.
doi:10.1371/journal.pone.0091512.t002

Measurement of optical density in Western blots. Western blots were performed four times for each sample. The optical density (OD) was measured by the Image J 1.42q software and the OD of each marker protein was calibrated with the OD of β-actin. The final OD of each marker protein for every animal was the average of the values acquired from the four blots of each sample.

Statistical analysis. All experimental data are presented as mean±SD (standard deviation). The statistical analyses of data were performed by one-way ANOVA followed by Fisher's *post hoc* PLSD test with SPSS 16.0 software and $p<0.05$ was considered to be significant.

Result

There was no significant difference in weight among groups (data not shown). Statistical analyses were made among the QA,

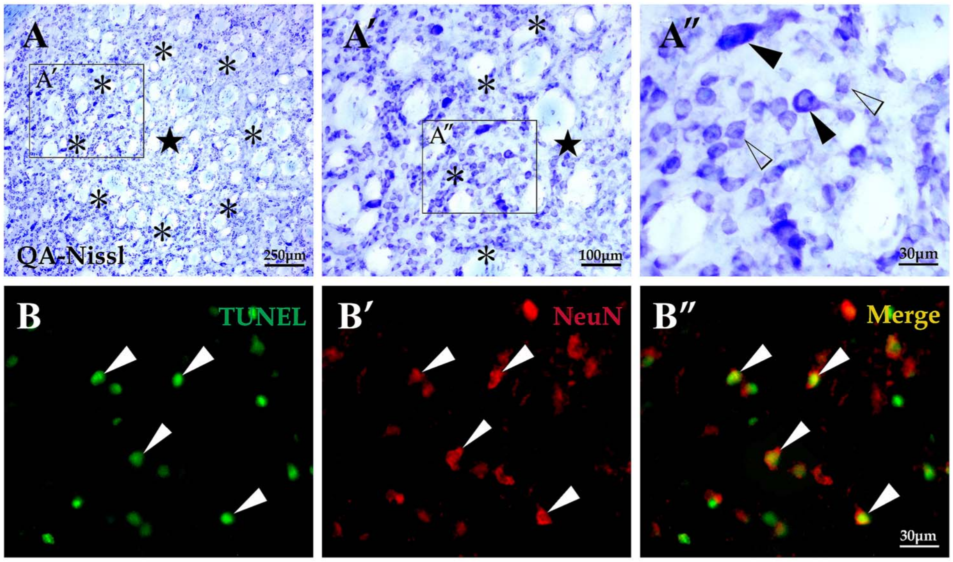

Figure 2. Histological changes and neuronal apoptosis of striatum induced by QA. In sections with Nissl staining taken from the QA-treated striatum (A-A″), the lesion core (★) was injured seriously with very few neurons survived; the annular area surrounded the lesion core, named the transition zone (*), was less injured than the lesion core with some neurons survived including several medium-sized neurons (white arrowheads in A″) and a few large-sized neurons (black arrowheads in A″); the periphery, the area outside the transition zone, appeared similar to tissues of a normal striatum. In sections with TUNEL labeling (B-B″), the apoptotic cells were mainly detected in the transition zone (white arrowheads in B) and a large proportion of them were neurons as shown in the TUNEL/NeuN double labeling section (white arrowheads in B″). Cont is short for control. Panels A′ and A″ are views of higher magnification from the boxes of Panels A and A′, respectively. Scale bars: A, 250 μm; A′, 100 μm; A″, 30 μm; B-B″, 30 μm.
doi:10.1371/journal.pone.0091512.g002

the control and the normal groups, and there was no significant difference between the control group and the normal group in both behavioral tests and histological examinations. To make the following report concise and easier to understand, we provide data and statistical analyses of the normal group and the control group (Table 1) before we reveal all of our findings. In the following text, we focus on the QA group and the control group.

Motor and cognitive deficits induced by QA

To assess the motor functions of experimental rats, the balance beam test and the grip strength test were applied. In the balance beam test, one QA-treated rat failed to pass across the beam, but the other nine cases in the QA group had difficulty in initiating movement and passing across the beam in stark contrast to the control rats (Fig. 1A and A′). The number of paw slipping ($F = 7.687$, $df1 = 2$, $df2 = 26$, $p<0.01$) and the completion time ($F = 12.905$, $df1 = 2$, $df2 = 26$, $p<0.01$) in the QA group were significantly increased when compared with the control group (Table 2). In the grip strength test, rats with intrastriatal QA injections showed hypertonia with longer hang time in comparison to the control rats ($F = 6.542$, $df1 = 2$, $df2 = 27$, $p<0.01$; Fig. 1B and B′; Table 2).

The cognitive and mnemonic deficits of experimental animals were detected by the water maze task. There was no significant difference among the QA, the control and the normal groups in swim speed (QA 17.25±5.89 cm/s, Cont 19.42±10.03 cm/s, Norm 18.52±8.01 cm/s; $F = 0.180$, $df1 = 2$, $df2 = 27$, $p>0.05$), and therefore the results reflecting cognitive functions should not have been affected by motor dysfunction in the task. QA-treated rats swam in bending routes when locating the hidden platform and often moved along the wall of the maze (Fig. 1C′). The latency of locating the platform was significantly increased in the learning trials ($F = 13.231$, $df1 = 2$, $df2 = 27$, $p<0.01$) and the number of target site crossovers was markedly decreased in the probe trial in the QA group ($F = 42.473$, $df1 = 2$, $df2 = 27$, $p<0.01$) when compared with the control group (Table 2).

Loss of striatal projection neurons in the transition zone induced by QA

In the experimental rats treated with intrastriatal QA injections, a unilateral lesion was observed in the striatum and the exact locations were usually around the injection sites. Based on analyses of brain sections processed with Nissl staining, a severe neuron loss was observed in the lesion core and the mean lesion diameter through the center of lesion was 1.02±0.13 mm (Fig. 2A and A′). The annular area surrounded the lesion core is termed transition zone (0.24±0.03 mm in width) [30] in which a relatively slighter neuron loss was observed. Among the remaining cells in this zone were a few large neurons mixed with some medium-sized ones (Fig. 2A″). The transition zone has been of great interest to researchers and clinicians due to its uniqueness of cell survival and similarity to pathophysiology of HD [25,30]. Thus, we mainly focused on the transition zone and performed cell count in this area. The cell abundance of the transition zone in QA-treated rats was significantly reduced in comparison of the control animals ($F = 21.772$, $df1 = 2$, $df2 = 12$, $p<0.01$; Fig. 3A). Outside the transition zone, cells that made up a region named periphery appeared indistinguishable from those in a normal striatum in terms of quantity and appearance.

To investigate whether apoptosis participated in neuron death in the QA-induced striatal impairment, the TUNEL assay was applied in the present study. In the QA group, TUNEL-labeled cellular fragments and pyknosis were mainly found in the transition zone (371.67±40.08/mm^2; Fig. 2B). Further double-labeling of TUNEL and NeuN displayed that a large proportion of cells going through apoptosis were NeuN+ neurons

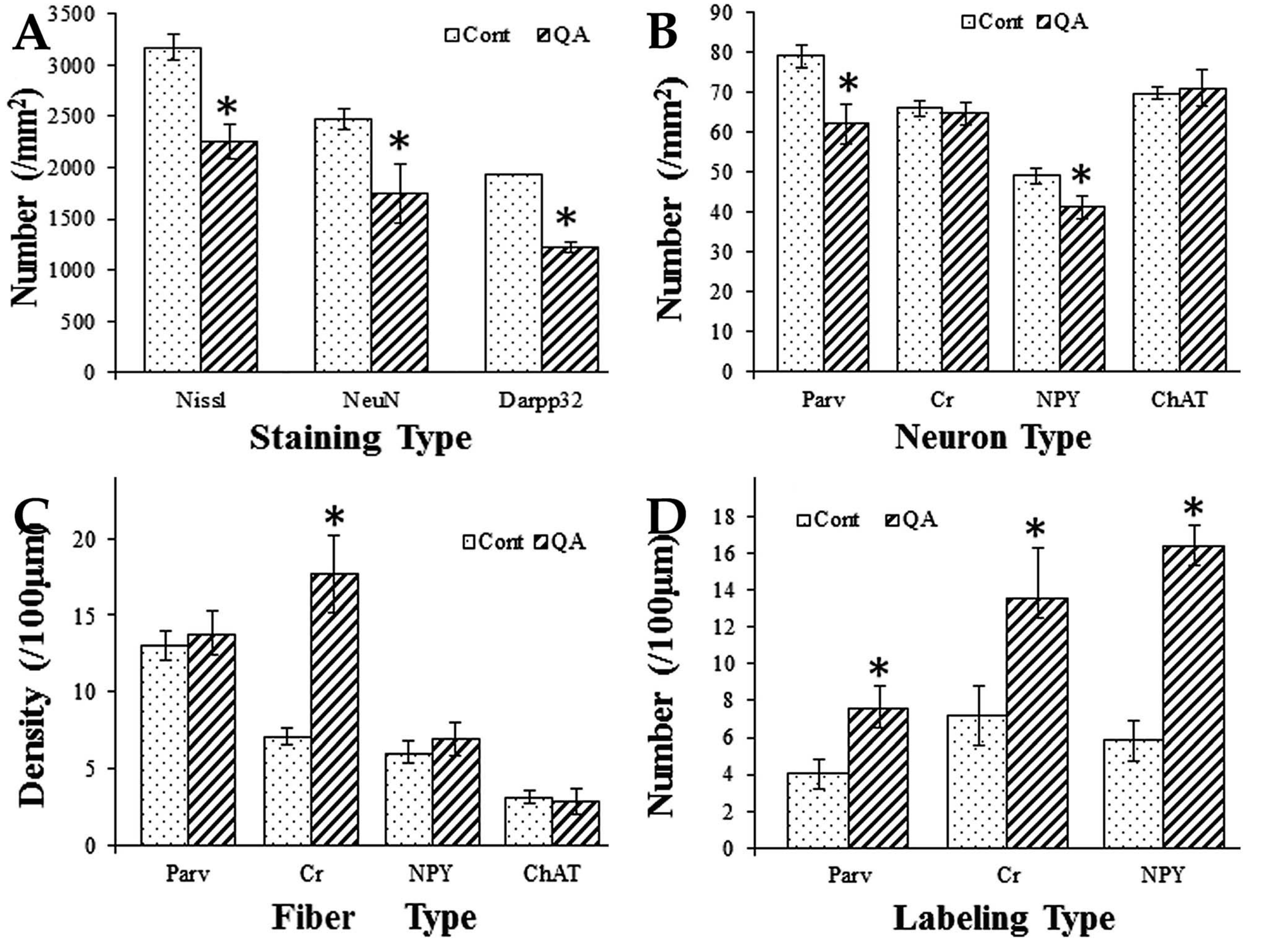

Figure 3. Histograms for comparison between the QA group and the control group. Histogram A was based on the statistical analyses of brain sections with Nissl staining as well as NeuN and Darpp32 immunolabeling. It revealed that the numbers of neurons and projection neurons in the transition zone were significantly decreased in the QA group when compared to the control group. Histograms B and C were based on the statistical analyses of brain sections with Parv, Cr, NPY and ChAT immunolabeling. The numbers of Parv+ and NPY+ interneurons were significantly decreased while those of Cr+ and ChAT+ interneurons were not notably reduced in the transition zone of the QA group when compared to the control group (B). The density of Cr+ fibers was significantly increased while those of Parv+, NPY+ and ChAT+ fibers were not notably changed in the transition zone of the QA group in comparison of the control group (C). Histogram D was based on the statistical analyses of brain sections with Parv, Cr and NPY immunolabeling. It revealed that the varicosity densities of Parv+, Cr+ and NPY+ fibers were all significantly increased in the transition zone of the QA group when compared to the control group. Values are expressed as group means±SD. * $P<0.01$ v.s. control, Student's *t*-test.
doi:10.1371/journal.pone.0091512.g003

(213.67±46.61/mm^2; Fig. 2B″). No TUNEL labeling was observed in control and normal rats.

Histologically, the striatum consists of a vast majority of projection neurons and a small number of interneurons [16]. We applied the antibody of NeuN labeling all the neurons and the antibody of Darpp32 specific for striatal projection neurons to assess the degree of neuron loss in the QA-treated striatum. Based on the analyses of brain sections with NeuN labeling, there was a significant difference in the number of neurons in the transition zone between QA-treated rats and control rats ($F = 24.005$, $df1 = 2$, $df2 = 12$, $p<0.01$; Fig. 3A; Fig. 4A, A′, B and B′). In sections with Darpp32 labeling, the number of projection neurons in the transition zone was significantly decreased in the QA group when compared with the control group ($F = 170.562$, $df1 = 2$, $df2 = 12$, $p<0.01$; Fig. 3A; Fig. 4C, C′, D and D′).

Changes of striatal interneurons in the transition zone induced by QA

By means of immunohistochemistry, the four interneuron types specifically labeled by antibodies of Parv, Cr, NPY and ChAT were investigated in the transition zone. The numbers of Parv+ interneurons ($F = 40.717$, $df1 = 2$, $df2 = 12$, $p<0.01$; Fig. 3B; Fig. 5A and B) and NPY+ interneurons ($F = 22.846$, $df1 = 2$, $df2 = 12$, $p<0.01$; Fig. 3B; Fig. 6A and B) were significantly reduced in the QA group in comparison to the control group. In contrast to the vulnerability of Darpp32+ projection neurons as well as Parv+ and NPY+ interneurons, the Cr+ interneurons and ChAT+ interneurons were relatively resistant to excitotoxicity induced by QA. The statistical analyses showed that there was no significant difference between the QA group and the control group in the numbers of Cr+ neurons ($F = 0.523$, $df1 = 2$, $df2 = 12$, $p>0.05$; Fig. 3B; Fig. 5C and D) and ChAT+ neurons ($F = 0.347$, $df1 = 2$, $df2 = 12$, $p>0.05$; Fig. 3B; Fig. 6C and D).

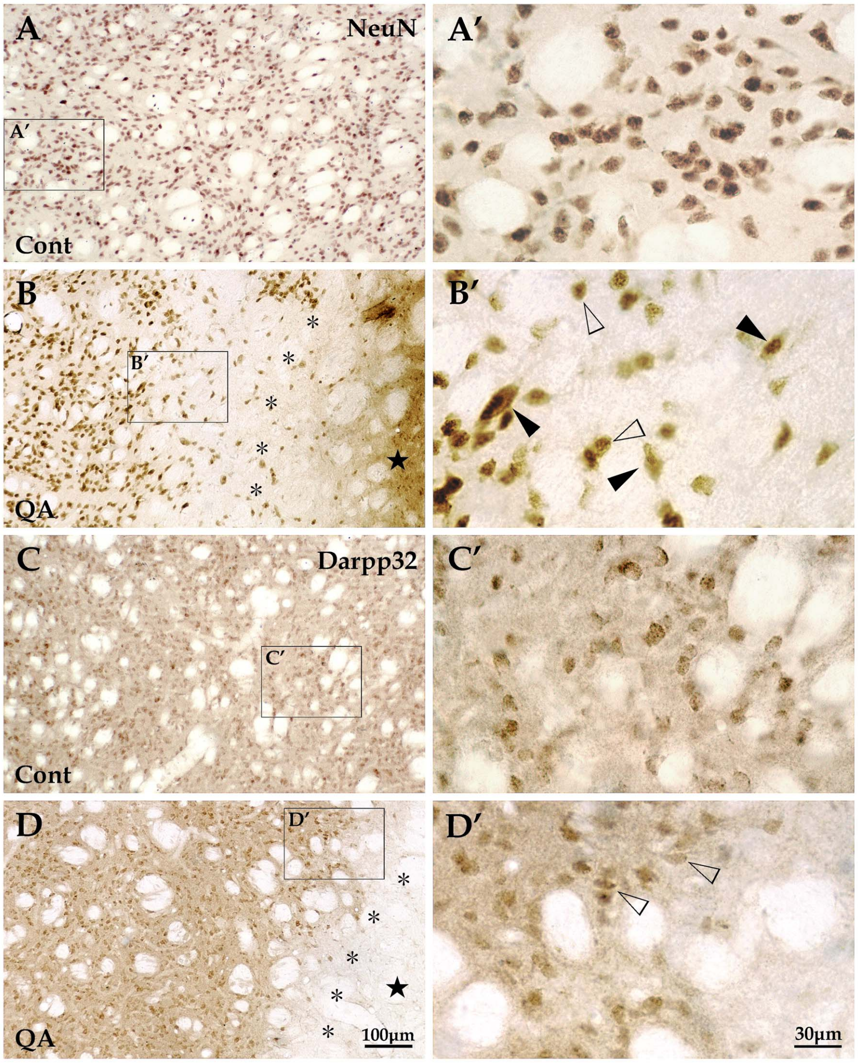

Figure 4. Changes of striatal projection neurons induced by QA. Striatal neurons, presented with NeuN labeling, were evenly distributed throughout the striatum in the control group (A and A′) while the NeuN+ cells in the QA group (B and B′) were extremely scarce in the lesion core (★) and notably reduced in quantity in the transition zone (*). Those neurons survived in the transition zone (B′) included some medium-sized neurons (white arrowheads) and a few large-sized neurons (black arrowheads). Striatal projection neurons, labeled for Darpp32, were medium in size and evenly distributed in the striatum in control rats (C and C′). The Darpp32+ projection neurons hardly survived in the lesion core and only a small proportion did in the transition zone (D and D′). Cont is short for control. Panels A′–D′ are views of higher magnification from the boxes of Panels A–D, respectively. Scale bars: A–D, 100 μm; A′–D′, 30 μm.
doi:10.1371/journal.pone.0091512.g004

In addition to changes of neuron abundance, the interneurons in the transition zone displayed morphological changes against QA injury. Though the neuron number was not notably reduced after QA treatment, Cr+ interneurons showed an obvious increase in fiber density when compared to the controls ($F = 83.145$, $df1 = 2$, $df2 = 12$, $p<0.01$; Fig. 3C; Fig. 5C and D′′). However, the fiber densities of Parv+, NPY+ and ChAT+ interneurons were not significantly increased in the QA group in comparison of the controls (Parv+ $F = 0.873$, $df1 = 2$, $df2 = 12$, $p>0.05$; NPY+ $F = 2.069$, $df1 = 2$, $df2 = 12$, $p>0.05$; ChAT+ $F = 0.450$, $df1 = 2$, $df2 = 12$, $p>0.05$; Fig. 3C; Fig. 5A′ and B′; Fig. 6A′–D′).

Moreover, the striatal GABAergic interneurons in the transition zone after QA treatment showed formation of varicosities along the neuronal processes. The varicosity densities of Parv+, Cr+ and NPY+ interneurons were notably increased in the QA group when compared with the controls (Parv+ $F = 21.322$, $df1 = 2$, $df2 = 12$, $p<0.01$; Cr+ $F = 17.959$, $df1 = 2$, $df2 = 12$, $p<0.01$; NPY+ $F = 160.422$, $df1 = 2$, $df2 = 12$, $p<0.01$; Fig. 3D; Fig. 5A′–D′; Fig. 6A′ and B′). Unlike the three types of GABAergic interneurons, the ChAT+ interneurons remained morphologically stable without varicosity formation (Fig. 6C′ and D′).

Marker protein changes of different striatal neurons induced by QA

To further detect changes of different striatal neurons, Western blots were applied in the present study. In line with the results from cell counts, the expression levels of NeuN ($F = 153.906$, $df1 = 2$, $df2 = 12$, $p<0.01$), Darpp32 ($F = 89.508$, $df1 = 2$, $df2 = 12$, $p<0.01$), Parv ($F = 170.509$, $df1 = 2$, $df2 = 12$, $p<0.01$) and NPY ($F = 42.763$, $df1 = 2$, $df2 = 12$, $p<0.01$) were significantly decreased while those of Cr ($F = 0.144$, $df1 = 2$, $df2 = 12$, $p>0.05$) and ChAT ($F = 0.675$, $df1 = 2$, $df2 = 12$, $p>0.05$) were not notably changed in the QA group when compared to the controls (Fig. 7).

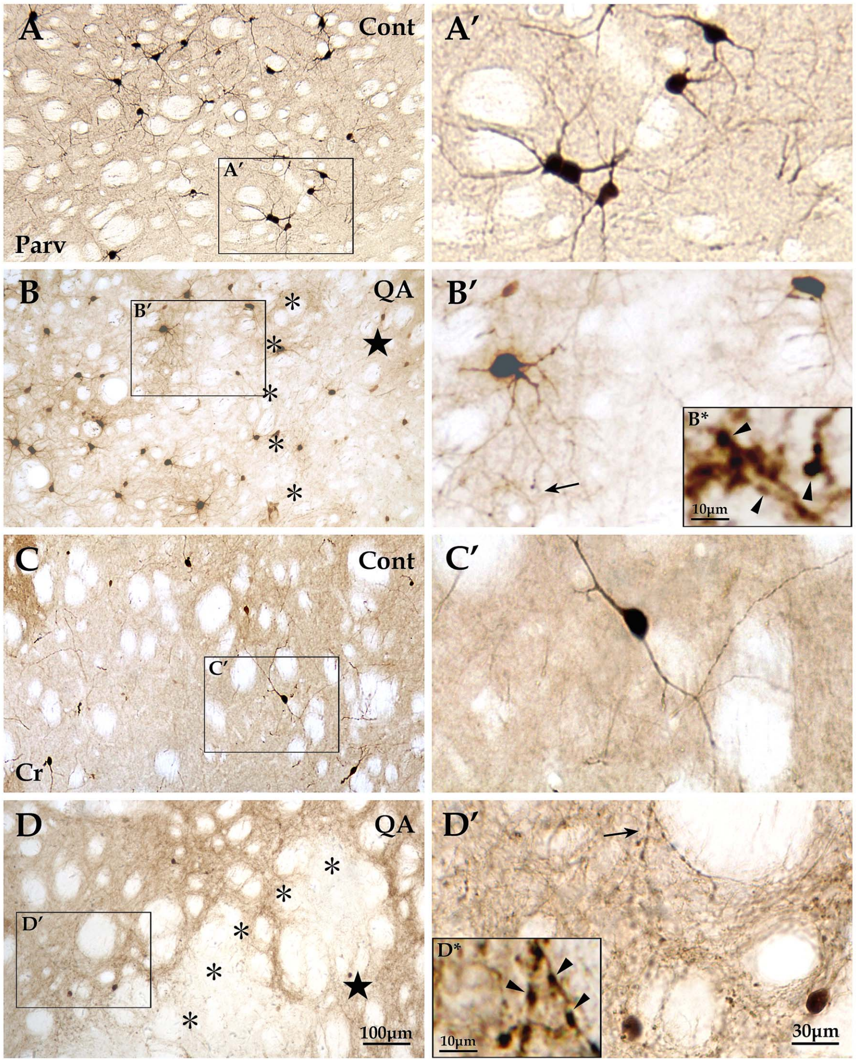

Figure 5. Reactions of Parv+ and Cr+ interneurons to QA. The Parv+ interneurons were mainly distributed in the dorsolateral striatum in the control group (A and A′). These interneurons in the QA group (B and B′) were extremely scarce in the lesion core (★) and presented some changes in the transition zone (*) including decrease in neuron number and increase in the number of varicosities which formed along the neuronal processes (see the arrow in B′ and the view of higher magnification in B*), but hyperplasia of fibers was not obvious. The Cr+ interneurons were mainly distributed in the medial striatum in the control rats (C and C′). In the QA group (D and D′), this interneuron type hardly survived in the lesion core but the neuron number was not notably reduced in the transition zone. However, the Cr+ interneurons reacted to QA by remarkably proliferating fibers and forming varicosities (see the arrow in D′ and the view of higher magnification in D*). Panels A′–D′ are views of higher magnification from the boxes of Panels A–D, respectively. Cont is short for control. Scale bars: A–D, 100 μm; A′–D′, 30 μm; B* and D*, 10 μm.
doi:10.1371/journal.pone.0091512.g005

Discussion

Behavioral impairments in QA-treated rats

HD is a neurodegenerative condition characterized by progressive abnormal involuntary movements (chorea, dyskinesia and dystonia) and cognitive impairment associated with perseverative behavior and impairment in strategy and planning [1,10]. Some specific neurotoxins such as QA and 3NP as well as transgenic models are used to study the pathophysiology of HD [9,10,31]. QA has long been utilized to induce HD models in rodents since it produces behavioral defects reminiscent of the human HD [9,32] and recent studies suggest that it is an endogenous metabolite of the kynurenine pathway (KP) which is critically involved in HD [12,33,34]. Previous research revealed that animals with QA treatment presented hyperactivity, significant impairment in the balance beam test and spatial learning deficits in the radial arm water maze task [17,22]. In line with these studies, the present study detected that QA-treated rats showed motor deficits in the balance beam test and cognitive impairment in the water maze task. Furthermore, our results revealed that QA treatment induced hypertonia in the grip strength test. Several studies have reported an increase of muscular tension by the grip strength test in animals with 3NP treatment [17,25]. Shear *et al* has suggested that QA treatment produced milder behavioral effects that mimicked some of the earlier symptoms of HD, while 3NP produced more severe effects which mimicked both the later symptoms and the juvenile onset of HD [17].

Pathological pattern and existence of apoptosis in QA-treated striatum

In human and rat brains, QA is present at concentrations in the high nanomolar range (usually less than 100 nM) [35,36], but it

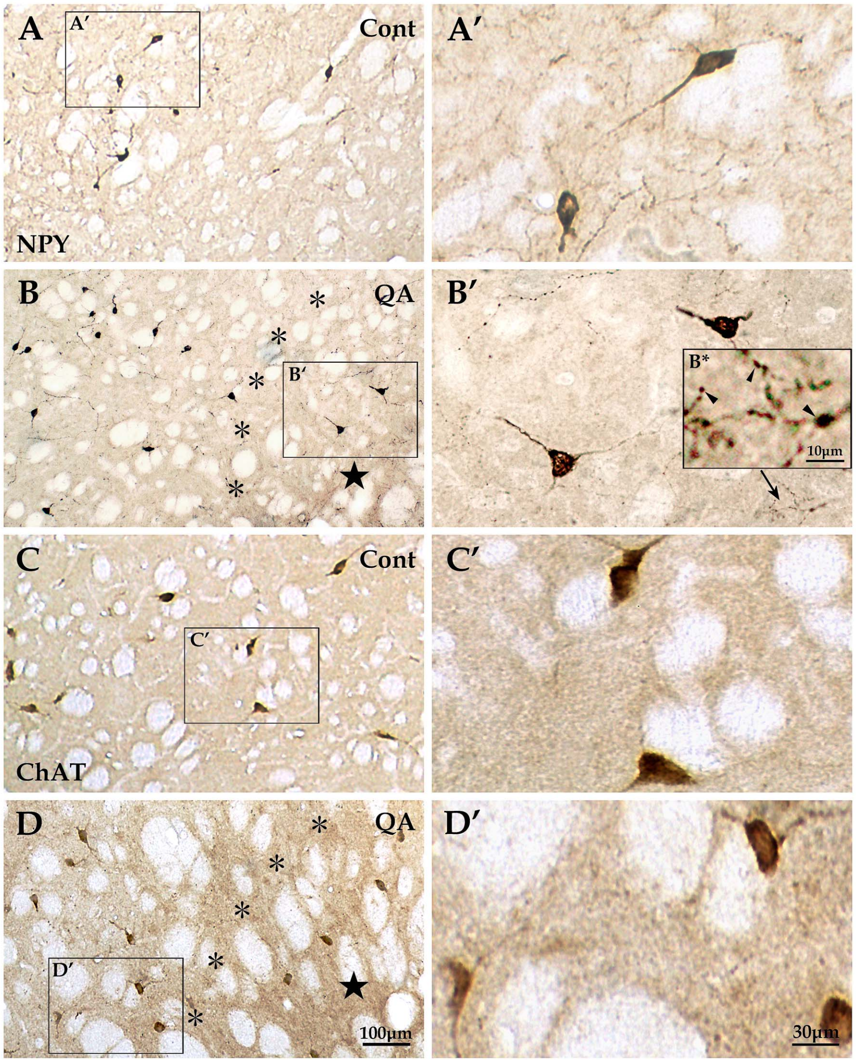

Figure 6. Reactions of NPY+ and ChAT+ interneurons to QA. The NPY+ interneurons were evenly distributed in the striatum in the control group (A and A′). This interneuron type in QA-treated rats (B and B′) hardly survived in the lesion core (★) and experienced some changes in the transition zone (*) including decrease in neuron number and increase in the number of varicosities which formed along the neuronal processes (see the arrow in B′ and the view of higher magnification in B*), but hyperplasia of fibers was not obvious. The ChAT+ interneurons were large in size and evenly distributed throughout the striatum in the control rats (C and C′) and they appeared stable in neuron abundance as well as in morphology without hyperplasia of fibers or varicosity formation in the QA-treated rats (D and D′). Panels A′–D′ are views of higher magnification from the boxes of Panels A–D, respectively. Cont is short for control. Scale bars: A–D, 100 µm; A′–D′, 30 µm; B*, 10 µm.
doi:10.1371/journal.pone.0091512.g006

has been reported that the brain level of QA was increased three to four-fold in low-grade HD brain [37]. Braidy *et al* showed that QA mediated astrocytic and neuronal inflammation and damage at sub-physiological concentrations (150 nM) *in vitro* [38]. However, the *in vivo* HD animal model required intrastriatal injections of QA at much higher concentrations (50–225 mM) to produce neuronal damage [19,20,39], as it did in the present study (100 mM). The reason may be that QA acts on the brain in an acute and focal manner in the *in vivo* model while it exerts prolonged and extensive effect on the neurons during the disease progression or in the *in vitro* experiments. Thus it requires a larger amount of the excitotoxin in the animal model to produce the similar effect as it does in the pathophysiologic state.

The pathological hallmark of HD is a massive reduction of the striatal volume which results from the loss of spiny projection neurons, while the aspiny interneurons are relatively spared [40]. It has been suggested that QA can produce axon-sparing lesions closely resembling those observed in HD [14,32]. In particular, the transition zone of QA-lesioned striatum has been of great interest to researchers due to its similarity to the pathophysiology of HD [30]. The present study did detect the selective neuron death in the transition zone rather than in the lesion core where there was hardly any striatal neuron survived. The underlying mechanisms of this selective sparing of neurons are not fully elucidated. Now growing amount of evidence supports the existence of apoptosis in HD human brains [41,42,43]. Accordingly, our data found that apoptosis was a major type of neuron death in the transition zone while necrosis was the main type of neuron death in the lesion core. Research on HD animal models and postmortem tissues has also pointed that the caspase family was shown to cleave mutant htt which is closely related to the pathogenesis of HD [44,45,46]. It is well-known that the excitotoxin QA exerts its toxic effects by

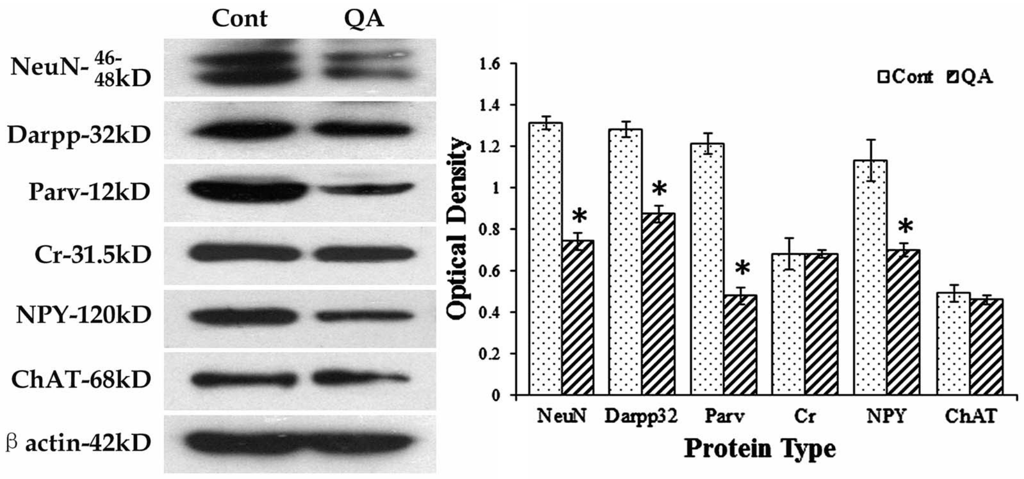

Figure 7. Western blots for marker proteins of different striatal neurons. Western blots were applied to detect the expression levels of NeuN, Darpp32, Parv, Cr, NPY and ChAT after QA treatment. β-actin was used to control for equal protein loading. In the histogram, levels indicated in the bars are expressed as optical density (OD). The ODs of NeuN, Darpp32, Parv and NPY were significantly decreased while those of Cr and ChAT were not notably changed in QA-treated striatum when compared to the controls (Cont). Values are expressed as group means±SD. * $P<0.01$ v.s. control, Student's *t*-test.
doi:10.1371/journal.pone.0091512.g007

overactivation of N-methyl-D-aspartate (NMDA) receptors and increased cytosolic Ca^{2+} concentrations [33]. In addition to the proven excitotoxic profile of QA, a considerable amount of evidence recently suggested that oxidative stress and energetic disturbances were major constituents of its toxic pattern [33,38,47]. Mitochondrial abnormalities, especially a deficit of energy metabolism in brains, have been reported in htt transgenic mice and it was associated with apoptotic cell death [48]. It has been proposed that prohibiting the activation process of apoptosis may be a potential neuroprotective therapy for HD [49].

Specific reactions of different striatal interneurons after QA treatment and their implications

The most intriguing findings in the present experiment were the specific reactions of different striatal interneuron types in the transition zone after QA treatment. In brief, our data showed that the Parv+ and NPY+ interneurons were relatively susceptible to QA toxicity, but to a lesser extent when compared to the projection neurons, while the Cr+ interneurons were relatively resistant and the ChAT+ interneurons seemed insensitive to QA treatment. Previous studies also revealed that the Parv+ and NPY+ striatal interneurons were relatively vulnerable to QA toxicity in rodents [17,20,50]. Figueredo-Cardenas *et al* further reported that the Parv+ neurons were relatively not impervious to QA while the NPY+ neurons were highly vulnerable in rats [51]. It is noteworthy that the Cr+ interneurons were of higher resistance to QA-mediated toxicity than the Parv+ and NPY+ neurons in the present experiment. Several chemical anatomical studies on striatal interneurons have reported that the Cr+ neurons were selectively spared in HD postmortem tissues [40,52,53]. Our assessment of neuron counts and Western blots also found that the number of Cr+ interneurons was not significantly decreased in QA-treated rats. The underlying mechanisms are still under exploration. It has been suggested that the calcium-binding protein Cr plays an important role in the maintenance of intracellular Ca^{2+} homeostasis. Its presence in some neurons may protect them against the massive Ca^{2+} entry that may result from over-stimulation on glutamate receptors [52].

The resistance of the GABAergic interneurons against QA toxicity was also manifested with morphological changes including hyperplasia of processes and varicosities. The present experiment showed that the fiber density of Cr+ neurons was markedly increased after QA treatment. Although the absolute fiber densities of Parv+ and NPY+ neurons were not significantly increased in QA-treated rats, the numbers of these two interneurons were reduced. Thus the fibers for each individual neuron were relatively increased. Though very few experiments have been conducted in this field, our previous studies on the 3NP-induced HD model have proven that the survival of NPY+ and Cr+ interneurons in the transition zone was accompanied by an increase of fibers and varicosities [25]. It has been proposed that this may be a response to the projection neuron loss, a protective reaction, or even one of the mechanisms to exacerbate the striatal neuron loss [25,54]. The three GABAergic interneurons directly modulate precise timing of action potential firing of projection neurons [55,56]. The great loss of projection neurons can be regarded as a strong stimulus to the survived interneurons where compensatory hyperplasia of fibers and varicosities occurs.

Unlike the three GABAergic interneuron types, our data revealed that the ChAT+ interneurons remained unchanged in abundance and morphology after QA treatment. Numerous studies have consistently indicated that the cholinergic interneurons were rather resistant or insensitive in HD postmortem striatum and animal models [50,57,58]. These may result from the attribute of autonomous electrical activity, or independence to synaptic input, which suggests that the ChAT+ neurons are relatively insensitive to glutamate-receptor-dependent excitotoxicity [59].

Author Contributions

Conceived and designed the experiments: QF SM WL. Performed the experiments: YM LO. Analyzed the data: JW SC. Contributed reagents/materials/analysis tools: WL. Wrote the paper: QF YM.

References

1. Walker FO (2007) Huntington's disease. Lancet 369: 218–228.
2. Ferrante RJ, Kowall NW, Beal MF, Martin JB, Bird ED, et al. (1987) Morphologic and histochemical characteristics of a spared subset of striatal neurons in Huntington's disease. J Neuropathol Exp Neurol 46: 12–27.
3. The Huntington's Disease Collaborative Research Group (1993) A novel gene containing a trinucleotide repeat that is expanded and unstable on Huntington's disease chromosomes. Cell 72: 971–983.
4. Albin RL, Tagle DA (1995) Genetics and molecular biology of Huntington's disease. Trends Neurosci 18: 11–14.
5. Claes S, Van Zand K, Legius E, Dom R, Malfroid M, et al. (1995) Correlations between triplet repeat expansion and clinical features in Huntington's disease. Arch Neurol 52: 749–753.
6. Kim SH, Thomas CA, Andre VM, Cummings DM, Cepeda C, et al. (2011) Forebrain striatal-specific expression of mutant huntingtin protein in vivo induces cell-autonomous age-dependent alterations in sensitivity to excitotoxicity and mitochondrial function. ASN Neuro 3: e60.
7. Cowan CM, Raymond LA (2006) Selective neuronal degeneration in Huntington's disease. Curr Top Dev Biol 75: 25–71.
8. Bithell A, Johnson R, Buckley NJ (2009) Transcriptional dysregulation of coding and non-coding genes in cellular models of Huntington's disease. Biochem Soc Trans 37: 1270–1275.
9. Schwarcz R, Guidetti P, Sathyasaikumar KV, Muchowski PJ (2010) Of mice, rats and men: Revisiting the quinolinic acid hypothesis of Huntington's disease. Prog Neurobiol 90: 230–245.
10. Brouillet E, Jacquard C, Bizat N, Blum D (2005) 3-Nitropropionic acid: a mitochondrial toxin to uncover physiopathological mechanisms underlying striatal degeneration in Huntington's disease. J Neurochem 95: 1521–1540.
11. Han I, You Y, Kordower JH, Brady ST, Morfini GA (2010) Differential vulnerability of neurons in Huntington's disease: the role of cell type-specific features. J Neurochem 113: 1073–1091.
12. Bruyn RP, Stoof JC (1990) The quinolinic acid hypothesis in Huntington's chorea. J Neurol Sci 95: 29–38.
13. Doble A (1999) The role of excitotoxicity in neurodegenerative disease: implications for therapy. Pharmacol Ther 81: 163–221.
14. Roberts RC, Ahn A, Swartz KJ, Beal MF, DiFiglia M (1993) Intrastriatal injections of quinolinic acid or kainic acid: differential patterns of cell survival and the effects of data analysis on outcome. Exp Neurol 124: 274–282.
15. Guillemin GJ (2012) Quinolinic acid, the inescapable neurotoxin. FEBS J 279: 1356–1365.
16. Durieux PF, Schiffmann SN, de Kerchove DA (2011) Targeting neuronal populations of the striatum. Front Neuroanat 5: 40.
17. Shear DA, Dong J, Gundy CD, Haik-Creguer KL, Dunbar GL (1998) Comparison of intrastriatal injections of quinolinic acid and 3-nitropropionic acid for use in animal models of Huntington's disease. Prog Neuropsychopharmacol Biol Psychiatry 22: 1217–1240.
18. Guyot MC, Hantraye P, Dolan R, Palfi S, Maziere M, et al. (1997) Quantifiable bradykinesia, gait abnormalities and Huntington's disease-like striatal lesions in rats chronically treated with 3-nitropropionic acid. Neuroscience 79: 45–56.
19. Ngai LY, Herbert J (2005) Glucocorticoid enhances the neurotoxic actions of quinolinic acid in the striatum in a cell-specific manner. J Neuroendocrinol 17: 424–434.
20. Figueredo-Cardenas G, Chen Q, Reiner A (1997) Age-dependent differences in survival of striatal somatostatin-NPY-NADPH-diaphorase-containing interneurons versus striatal projection neurons after intrastriatal injection of quinolinic acid in rats. Exp Neurol 146: 444–457.
21. Meade CA, Figueredo-Cardenas G, Fusco F, Nowak TJ, Pulsinelli WA, et al. (2000) Transient global ischemia in rats yields striatal projection neuron and interneuron loss resembling that in Huntington's disease. Exp Neurol 166: 307–323.
22. Haik KL, Shear DA, Schroeder U, Sabel BA, Dunbar GL (2000) Quinolinic acid released from polymeric brain implants causes behavioral and neuroanatomical alterations in a rodent model of Huntington's disease. Exp Neurol 163: 430–439.
23. Boegman RJ, Parent A (1988) Differential sensitivity of neuropeptide Y, somatostatin and NADPH-diaphorase containing neurons in rat cortex and striatum to quinolinic acid. Brain Res 445: 358–362.
24. MacKenzie GM, Jenner P, Marsden CD (1995) The effect of nitric oxide synthase inhibition on quinolinic acid toxicity in the rat striatum. Neuroscience 67: 357–371.
25. Mu S, OuYang L, Liu B, Zhu Y, Li K, et al. (2011) Preferential interneuron survival in the transition zone of 3-NP-induced striatal injury in rats. J Neurosci Res 89: 744–754.
26. Paxinos G, Watson C (2006) The rat brain in stereotaxic coordinates. United States: ACADEMIC PRESS. 1–456 p.
27. Vorhees CV, Williams MT (2006) Morris water maze: procedures for assessing spatial and related forms of learning and memory. Nat Protoc 1: 848–858.
28. Packard MG, Knowlton BJ (2002) Learning and memory functions of the Basal Ganglia. Annu Rev Neurosci 25: 563–593.
29. Voogd J, Feirabend HKP (1981) Methods in neurobiology, vol 2. New York: Elsevier. 301–364 p.
30. Huang Q, Zhou D, Sapp E, Aizawa H, Ge P, et al. (1995) Quinolinic acid-induced increases in calbindin D28k immunoreactivity in rat striatal neurons in vivo and in vitro mimic the pattern seen in Huntington's disease. Neuroscience 65: 397–407.
31. Bates GP, Mangiarini L, Mahal A, Davies SW (1997) Transgenic models of Huntington's disease. Hum Mol Genet 6: 1633–1637.
32. Beal MF, Kowall NW, Ellison DW, Mazurek MF, Swartz KJ, et al. (1986) Replication of the neurochemical characteristics of Huntington's disease by quinolinic acid. Nature 321: 168–171.
33. Perez-De LCV, Carrillo-Mora P, Santamaria A (2012) Quinolinic Acid, an endogenous molecule combining excitotoxicity, oxidative stress and other toxic mechanisms. Int J Tryptophan Res 5: 1–8.
34. Guidetti P, Schwarcz R (2003) 3-Hydroxykynurenine and quinolinate: pathogenic synergism in early grade Huntington's disease? Adv Exp Med Biol 527: 137–145.
35. Wolfensberger M, Amsler U, Cuenod M, Foster AC, Whetsell WJ, et al. (1983) Identification of quinolinic acid in rat and human brain tissue. Neurosci Lett 41: 247–252.
36. Braidy N, Grant R, Brew BJ, Adams S, Jayasena T, et al. (2009) Effects of Kynurenine Pathway Metabolites on Intracellular NAD Synthesis and Cell Death in Human Primary Astrocytes and Neurons. Int J Tryptophan Res 2: 61–69.
37. Guidetti P, Luthi-Carter RE, Augood SJ, Schwarcz R (2004) Neostriatal and cortical quinolinate levels are increased in early grade Huntington's disease. Neurobiol Dis 17: 455–461.
38. Braidy N, Grant R, Adams S, Brew BJ, Guillemin GJ (2009) Mechanism for quinolinic acid cytotoxicity in human astrocytes and neurons. Neurotox Res 16: 77–86.
39. Kalonia H, Kumar P, Kumar A (2011) Comparative neuroprotective profile of statins in quinolinic acid induced neurotoxicity in rats. Behav Brain Res 216: 220–228.
40. Massouh M, Wallman MJ, Pourcher E, Parent A (2008) The fate of the large striatal interneurons expressing calretinin in Huntington's disease. Neurosci Res 62: 216–224.
41. Sawa A, Tomoda T, Bae BI (2003) Mechanisms of neuronal cell death in Huntington's disease. Cytogenet Genome Res 100: 287–295.
42. Dragunow M, Faull RL, Lawlor P, Beilharz EJ, Singleton K, et al. (1995) In situ evidence for DNA fragmentation in Huntington's disease striatum and Alzheimer's disease temporal lobes. Neuroreport 6: 1053–1057.
43. Thomas LB, Gates DJ, Richfield EK, O'Brien TF, Schweitzer JB, et al. (1995) DNA end labeling (TUNEL) in Huntington's disease and other neuropathological conditions. Exp Neurol 133: 265–272.
44. Portera-Cailliau C, Hedreen JC, Price DL, Koliatsos VE (1995) Evidence for apoptotic cell death in Huntington disease and excitotoxic animal models. J Neurosci 15: 3775–3787.
45. Sanchez MR, Friedlander RM (2001) Caspases in Huntington's disease. Neuroscientist 7: 480–489.
46. Hermel E, Gafni J, Propp SS, Leavitt BR, Wellington CL, et al. (2004) Specific caspase interactions and amplification are involved in selective neuronal vulnerability in Huntington's disease. Cell Death Differ 11: 424–438.
47. Tasset I, Perez-De LCV, Elinos-Calderon D, Carrillo-Mora P, Gonzalez-Herrera IG, et al. (2010) Protective effect of tert-butylhydroquinone on the quinolinic-acid-induced toxicity in rat striatal slices: role of the Nrf2-antioxidant response element pathway. Neurosignals 18: 24–31.
48. Tabrizi SJ, Workman J, Hart PE, Mangiarini L, Mahal A, et al. (2000) Mitochondrial dysfunction and free radical damage in the Huntington R6/2 transgenic mouse. Ann Neurol 47: 80–86.
49. Tarawneh R, Galvin JE (2010) Potential future neuroprotective therapies for neurodegenerative disorders and stroke. Clin Geriatr Med 26: 125–147.
50. Meade CA, Figueredo-Cardenas G, Fusco F, Nowak TJ, Pulsinelli WA, et al. (2000) Transient global ischemia in rats yields striatal projection neuron and interneuron loss resembling that in Huntington's disease. Exp Neurol 166: 307–323.
51. Figueredo-Cardenas G, Harris CL, Anderson KD, Reiner A (1998) Relative resistance of striatal neurons containing calbindin or parvalbumin to quinolinic acid-mediated excitotoxicity compared to other striatal neuron types. Exp Neurol 149: 356–372.
52. Cicchetti F, Prensa L, Wu Y, Parent A (2000) Chemical anatomy of striatal interneurons in normal individuals and in patients with Huntington's disease. Brain Res Brain Res Rev 34: 80–101.
53. Cicchetti F, Gould PV, Parent A (1996) Sparing of striatal neurons coexpressing calretinin and substance P (NK1) receptor in Huntington's disease. Brain Res 730: 232–237.
54. Kawaguchi Y (1993) Physiological, morphological, and histochemical characterization of three classes of interneurons in rat neostriatum. J Neurosci 13: 4908–4923.
55. Tepper JM, Tecuapetla F, Koos T, Ibanez-Sandoval O (2010) Heterogeneity and diversity of striatal GABAergic interneurons. Front Neuroanat 4: 150.
56. Tepper JM, Bolam JP (2004) Functional diversity and specificity of neostriatal interneurons. Curr Opin Neurobiol 14: 685–692.

57. Smith R, Chung H, Rundquist S, Maat-Schieman ML, Colgan L, et al. (2006) Cholinergic neuronal defect without cell loss in Huntington's disease. Hum Mol Genet 15: 3119–3131.
58. Davies SW, Roberts PJ (1988) Sparing of cholinergic neurons following quinolinic acid lesions of the rat striatum. Neuroscience 26: 387–393.
59. Goldberg JA, Reynolds JN (2011) Spontaneous firing and evoked pauses in the tonically active cholinergic interneurons of the striatum. Neuroscience 198: 27–43.
